# *In Silico *screening for functional candidates amongst hypothetical proteins

**DOI:** 10.1186/1471-2105-10-289

**Published:** 2009-09-16

**Authors:** Claus Desler, Prashanth Suravajhala, May Sanderhoff, Merete Rasmussen, Lene Juel Rasmussen

**Affiliations:** 1 Department of Science, Systems and Models, Roskilde University, DK-4000 Roskilde, Denmark; 2 Centre for Development of Advanced Computing - Bioinformatics Team, Scientific and Engineering Computing Group, Pune University Campus, Pune 411007, India; 3 Center for Healthy Aging, Faculty of Health Sciences, Copenhagen University, DK-2200 Copenhagen N, Denmark

## Abstract

**Background:**

The definition of a hypothetical protein is a protein that is predicted to be expressed from an open reading frame, but for which there is no experimental evidence of translation. Hypothetical proteins constitute a substantial fraction of proteomes of human as well as of other eukaryotes. With the general belief that the majority of hypothetical proteins are the product of pseudogenes, it is essential to have a tool with the ability of pinpointing the minority of hypothetical proteins with a high probability of being expressed.

**Results:**

Here, we present an *in silico *selection strategy where eukaryotic hypothetical proteins are sorted according to two criteria that can be reliably identified *in silico*: the presence of subcellular targeting signals and presence of characterized protein domains. To validate the selection strategy we applied it on a database of human hypothetical proteins dating to 2006 and compared the proteins predicted to be expressed by our selecting strategy, with their status in 2008. For the comparison we focused on mitochondrial proteins, since considerable amounts of research have focused on this field in between 2006 and 2008. Therefore, many proteins, defined as hypothetical in 2006, have later been characterized as mitochondrial.

**Conclusion:**

Among the total amount of human proteins hypothetical in 2006, 21% have later been experimentally characterized and 6% of those have been shown to have a role in a mitochondrial context. In contrast, among the selected hypothetical proteins from the 2006 dataset, predicted by our strategy to have a mitochondrial role, 53-62% have later been experimentally characterized, and 85% of these have actually been assigned a role in mitochondria by 2008.

Therefore our *in silico *selection strategy can be used to select the most promising candidates for subsequent *in vitro *and *in vivo *analyses.

## Background

According to the Human Genome Organization (HUGO), the human genome is predicted to consist of 19599 protein-encoding genes [[1], Human Genome Project ]. A substantial part of these genes is predicted to encode a group of proteins, where translation has not been demonstrated and the proteins themselves have not been characterized. This group of proteins is accordingly defined as hypothetical. Although many of the listed hypothetical proteins most likely are predicted products of pseudogenes, there is a reasonable probability that a number of the listed hypothetical proteins are truly novel and can perform uncharacterized biological functions. Consequently, the putative importance of hypothetical proteins is not negligible.

Several *in silico *methods are available for descriptive predictions of proteins with unknown function. These include studies of homology, database searches for orthologs, or the presence of characterized functional domains or motifs within the protein [2]. Most often false positives will occur and predictions must be substantiated by *in vitro *and/or *in vivo *experiments to validate and further characterize predicted functionality. The *in silico *methods are designed for functional prediction of a protein, but not specifically designed to ascertain whether a protein is hypothetical or not. When screening hypothetical proteins for novel translatable candidates, *in silico *methods are therefore rarely used and the researcher often performs the screen with laborious *in vitro *and/or *in vivo *experiments.

In the present study, we propose an *in silico *screening strategy for eukaryotic systems, in which novel translatable candidates can be selected from a group of hypothetical proteins. The strategy is based on *in silico *methods normally used to make functional predictions of proteins, which include search for presence of sub-cellular targeting signals and for presence of characterized protein domains. Especially targeting signals and, to a lesser extent, protein domains can be predicted with high probability. The occurrence of either targeting signals or identifiable protein domains can also be present in pseudogenes as a result of gene duplication. However, we hypothesize that the risk of a hypothetical protein being a pseudogene is greatly reduced when both targeting signals and protein domains are identified in the transcript, especially if the protein domain architecture suggests a relevant function in the predicted sub-cellular compartment. Selection of hypothetical proteins based on a combination of both these factors should therefore greatly increase the success rate of discovering true functional proteins with roles in subcellular compartments among hypothetical proteins. Due to the design of the selection strategy it is ineffective for identifying proteins without localization signals, and this must be taken into consideration.

To exemplify our selection strategy we have chosen mitochondria as the targeted sub-cellular compartment. Within recent years, a substantial amount of work has been invested in compiling a near complete list of mitochondrial proteins in humans. This has resulted in the establishment of the MitoCarta database [3]. The total number of genes encoding mitochondrial proteins is according to MitoCarta at least 1013 [3]. Mitochondria are semiautonomous organelles present in almost all eukaryotic cells ranging from a single copy to several thousands. Mitochondria contain their own autonomous genome, which encodes 37 of these proteins. The remainder is encoded by nuclear DNA and imported into mitochondria. Examples of mitochondrial functions include ATP production by oxidative phosphorylation, β-oxidation of fatty acids, metabolism of amino acids and of lipids. Furthermore, mitochondria have a prominent role in apoptosis.

With the exception of proteins encoded by the mitochondrial genome, proteins are translated in the cytosol from their corresponding mRNA. Many proteins are transported to specific parts of the cell where they function in context of the sub-cellular compartment. The sub-cellular localization of proteins can be facilitated by specific targeting peptides. There are two types of targeting peptides, the presequences and the internal targeting signals. Presequences are often localized at the N-terminal whereas internal targeting signals can be distributed throughout the protein sequence [4-6]. The mitochondrial membrane contains translocases for recognition and import of nuclear-encoded mitochondrial proteins. The translocase of the outer mitochondrial membrane (TOM complex) is responsible for recognition and initial import of nuclear-encoded mitochondrial proteins (reviewed in [7]). Mitochondrial precursor proteins posses either an N-terminal presequence or internal targeting signals. Both types of targeting peptides, N-terminal or internal, are recognized by different import receptors of the TOM complex. N-terminal presequences generally have a length of 6-85 amino acid residues, enriched in Arg, Ser and Ala, while negatively charged amino acids are rarely present [8]. N-terminal presequences form positively charged amphiphilic α-helices when bound to import receptors on the mitochondrial surface [9], and upon mitochondrial import, presequences are removed by proteolysis (reviewed in [10,11]). Even though binding of different parts of the TOM complex to varying internal targeting signals has been shown [12], a common motif for an internal targeting signal still has to be elucidated,

In order to validate that our *in silico *selection strategy can predict functional candidates among hypothetical proteins we chose to focus on proteins with a predicted mitochondrial function. We have utilized an existing database of hypothetical proteins assembled in 2006 [13]. From this database we selected all hypothetical proteins predicted to be localized in human mitochondria due to the presence of a putative mitochondrial N-terminal presequence. These selected proteins were then investigated for the presence of potentially functional protein domains. We predict that the sub-group of hypothetical proteins, with both a mitochondrial N-terminal presequence and potentially functional protein domains has a high probability of being expressed and of having a function in a mitochondrial context.

All proteins investigated were hypothetical in 2006. However, between 2006 and 2008, many proteins have been experimentally characterized or removed from the database as they have been proven to be products of pseudogenes. This increases the probability that the 2006 dataset of hypothetical proteins includes a large number of proteins that are now (as of 2008) classified as mitochondrial. By applying the selection strategy on the 2006 dataset, we are able to compare the resulting predictions with the factual *in vitro *and/or *in vivo *characterizations of the proteins performed from 2006 to 2008. Effectiveness of the selection strategy can be demonstrated by comparing proteins selected from the 2006 dataset with the number of these proteins that, as of November 2008, are demonstrated to be translated, mitochondrial or proven to be pseudogenes.

## Methods

We have utilized a database of proteins extracted from GenBank in August 2006. At the time of extraction, all proteins were defined as hypothetical and all sequences were crosschecked and annotated [13]. In November 2008, the status of each individual protein was reinvestigated and entries of the 2006 database that later have been identified as duplicates were removed. The entries of the 2006 dataset were divided into three groups according to their individual status in November 2008: Hypothetical proteins, characterized proteins and proteins discovered to be pseudogenes and therefore removed by GenBank. These three groups are in the following collectively referred to as the 2008 dataset.

Several prediction programs have been designed to predict the localization of eukaryotic proteins. In table 1, we have listed a selection of available programs, which have been reported to have a medium to high prediction accuracy [14-22]. To exemplify the occurrence of hypothetical proteins with functional targeting peptides, hypothetical proteins from the 2006 dataset were analyzed using pTarget. The pTarget program  predicts protein targeting to nine different sub-cellular locations including mitochondria. Prediction is based on the occurrence of specific Pfam domains earlier determined to be location specific. pTarget, can predict 68-87% of the true positives at accuracy rates of 96-99% [19,20].

**Table 1:** Overview of subcellular localization prediction programs for eukaryotic proteins

**Classification method**	**Number of localization sites**	**Accuracy**
**BaCelLo **[14]	4-5	67-76%
**LOCtree **[15]	4	74%
**MITOPRED **[16]	1	85%
**MultiLoc **[17]	11	75%
**PA-SUB **[18]	11	81-94%
**pTarget **[19,20]	9	68-87%
**TargetP **[21]	3	90%
**WoLF PSORT **[22]	12	80%

A selection of subcellular localization prediction programs for eukaryotic proteins reported to have a medium to high prediction accuracy. Listed are the numbers of compartments each program can predict targeting to, and the reported accuracy of the prediction.

In contrast to pTarget, the TargetP program  predicts mitochondrial localization using the N-terminal sequence information only, with a success rate of predictions of 90% [21]. TargetP was used to screen the 2006 dataset for functional mitochondrial targeting peptides.

All hypothetical proteins predicted to have a mitochondrial targeting peptide by TargetP, were further characterized using the SMART program . The SMART program identifies protein domains from a database of manually annotated known protein domains [23,24].

We hypothesize that hypothetical proteins, predicted to contain both a mitochondrial N-terminal presequence and functional protein domains have a high probability of being functional in a mitochondrial context. To verify our hypothesis, we used the 2006 dataset of the, then, hypothetical proteins. Using TargetP, we selected proteins having a high probability of containing a mitochondrial N-terminal presequence. For the resulting subset of proteins we used SMART to search for the presence of functional protein domains. Comparing with protein status according to the 2008 dataset, we determined the percentage of selected proteins that had either been removed or experimentally characterized after 2006. Furthermore, if proteins had been experimentally characterized, we determined if they had been found to be functional in a mitochondrial context.

To demonstrate that the effectiveness of our *in silico *selection strategy is not dependent on neither TargetP nor the SMART program, the localization prediction programs, MITOPRED [16] and WoLF PSORT [22] were used in conjunction with the SMART program, to screen the 2006 dataset for proteins predicted to be mitochondrial. MITOPRED and WoLF PSORT have been reported to have high prediction accuracy of proteins that are localized to the mitochondria. Prediction of localization is based on the occurrence of Pfam domains and known sorting motifs rather than the presence of mitochondrial presequences as basis for prediction of protein targeting [16,22]. The effectiveness of the selection strategy using MITOPRED or WoLF PSORT was compared to the effectiveness of the selecting strategy using TargetP. Correspondingly, the effectiveness of our selection strategy was investigated, when the SMART program was replaced with the Prosite scanning tool [25]. Like the SMART program, Prosite identifies protein domains from a database of manually annotated known protein domains.

## Results and discussion

After removing proteins found or predicted to be duplicates of already existing proteins, the 2006 dataset of hypothetical proteins contains 5860 proteins. According to GenBank's current annotation (November 2008) of the same group of proteins, 1455 of the 5860 proteins annotated as hypothetical in 2006, are still hypothetical, while 1215 proteins have been experimentally characterized and 3190 proteins have been removed by GenBank as they have been identified as pseudogenes (See Additional file 1).

pTarget was used to predict the distribution of human hypothetical proteins from the 2006 dataset and the 2008 dataset (Table 2). pTarget is used to exemplify how human hypothetical proteins can be sorted based on their predicted cellular localization. This is important since our selection strategy is limited to proteins targeted for a sub-cellular localization. Using pTarget as an indicator only, we are able to demonstrate that proteins predicted to be localized to lysosomes, golgi, peroxysomes, mitochondria or endoplasmic reticulum, comprise of 32% of the 2006 dataset. When including proteins predicted to be secreted, proteins targeted for the plasma membrane or nucleus, these include 87% of the 2006 dataset. This indicates that the selection strategy, according to pTarget, can be applied on up to 87% of the dataset.

**Table 2:** Predicted subcellular distribution of human hypothetical proteins

**Compartment**	**Predicted subcellular distribution 2006 dataset (5860 proteins)**	**Predicted subcellular distribution 2008 dataset of hypothetical proteins (1455 proteins)**
Nucleus	37%	36%
Cytoplasm	13%	14%
Plasma membrane	12%	8%
Lysosomes	9%	9%
Golgi	9%	11%
Peroxysomes	7%	10%
Extracellular/Secretory	6%	4%
Mitochondria	5%	5%
Endoplasmic reticulum	2%	3%

The protein localization prediction program pTarget was used to predict the subcellular localization of 5860 and 1455 hypothetical proteins from the 2006 and 2008 datasets respectively.

TargetP predicts probability of mitochondrial localization based solely on mitochondrial specific presequences. These motifs do not necessarily require *cis *or *trans *acting domains in order to be fully functional mitochondrial targeting signals. Accordingly, if a hypothetical protein is predicted to be localized to the mitochondria, there is a reasonable probability that a corresponding, expressed protein would be localized to this organelle even though it may still be the product of a pseudogene. Screening the 2006 dataset of human hypothetical proteins with TargetP we found a total of 1139 proteins predicted to be localized to mitochondria (See Additional file 1). TargetP places all of the predicted proteins into reliability classes, ranging from A to E, where A indicates the strongest prediction [21]. We have focused on the total of 538 proteins belonging to reliability class A (52 proteins), B (204 proteins) and C (282 proteins) (See Additional file 1).

From reliability class A to C, 315 of 538 proteins (59%) selected by TargetP, have been removed, while 75 of 538 proteins (14%) have been characterized and 32 of 538 proteins (6%) have been characterized as mitochondrial. When focusing on proteins predicted by TargetP to be in reliability class A, 15 of 52 proteins (29%) have been removed, 18 of 52 proteins (35%) have been characterized and 14 of 52 proteins (27%) have been characterized as mitochondrial (See Additional file 1). From the 2006 dataset of hypothetical proteins, 67 are listed in MitoCarta as characterized mitochondrial proteins (See Additional file 1). TargetP was successful in identifying 32 of the total of 67 mitochondrial proteins, but did also select 315 proteins that after 2006 have been removed by GenBank. When focusing on proteins belonging to reliability class A, 14 of 67 mitochondrial proteins were identified, while 15 proteins have been removed. This demonstrates that TargetP is efficient in finding mitochondrial proteins, but it is not suitable for screening hypothetical proteins for novel translatable candidates. To be able to annotate the selected hypothetical proteins we screened proteins from reliability class A to C with the SMART program to determine the presence of known protein domains. We ignored proteins only containing transmembrane domains, coiled coil regions, signal peptides and/or segments of low compositional complexity, as these regions are not unique protein domains.

Three groups of proteins were constructed from the 538 proteins investigated. Group I consists of 20 proteins, predicted by TargetP to belong to reliability class A and to contain identifiable protein domains according to SMART prediction. Group II consists of 56 proteins, predicted by TargetP to belong to reliability classes A and B and to contain identifiable protein domains. Group III contains 100 proteins that are predicted by TargetP to belong to reliability classes A, B and C and to contain identifiable protein domains. Group III therefore contains all proteins of group I + II and group II contains all proteins of group I. The construction of these three groups allows us to comment on how reliable the prediction of cellular localization should be in order to get a good result from our selection strategy.

The three groups of selected proteins were, together with the 5860 hypothetical proteins from the 2006 dataset, compared with their corresponding 2008 annotations. The comparison includes number of experimentally characterized proteins, number of experimentally characterized mitochondrial proteins and number of proteins removed due to being the predicted result of pseudogenes or due to having similarity to an existing protein (Table 3).

**Table 3:** Comparison of predicted versus experimentally determined status of proteins

**Group**	**Localization signal/protein domain**	**Hypothetical proteins**	**Characterized proteins**	**Removed proteins**	**Characterized mitochondrial proteins**
**I**	20	30% (6 of 20)	65% (13 of 20)	5% (1 of 20)	85% (11 of 13)
**II**	56	27% (15 of 56)	64% (36 of 56)	9% (5 of 56)	58% (21 of 36)
**III**	100	36% (36 of 100)	53% (53 of 100)	11% (11 of 100)	45% (24 of 53)

**2006 dataset**	-	25% (1455 of 5860)	21% (1215 of 5860)	54% (3190 of 5860)	6% (67 of 1215)

Hypothetical proteins from the 2006 dataset sorted into groups depending on the probability of having a mitochondrial N-terminal presequence localization signal. Proteins of Group I, have been predicted by TargetP to belong to reliability class A, indicating the strongest prediction. Proteins of Group II contain proteins belonging to reliability class A and B, while proteins of Group III contain proteins belonging to reliability class A, B and C. All proteins of Group I, II and III have identifiable protein domains according to SMART. The three groups have been compared with all 5860 proteins of the 2006 dataset, and with their respective 2008 annotations, to evaluate whether the proteins have been characterized as being mitochondrial or have been removed.

25% of the 5860 proteins were in November 2008 still annotated as hypothetical, 21% had been experimentally characterized and 54% have been removed. Of the experimentally characterized proteins, 67 proteins or 6% were listed in MitoCarta as mitochondrial. Group III contains 100 proteins, where 36%, as of November 2008, are still hypothetical, 53% have been characterized and 11% have been removed. Of the characterized proteins 45% or 24 proteins are listed in MitoCarta as mitochondrial. The values obtained clearly demonstrate, that our strategy using a selection based both on the presence of a mitochondrial presequence and identifiable protein domains is very efficient for extracting hypothetical proteins with a functional role in mitochondria. Applying the selection strategy on the 2006 dataset identified 24 of 67 proteins that later have been categorized as mitochondrial. Furthermore the percentage of removed proteins is 5-fold lower for group III when compared with the 2006 dataset and the percentage of characterized proteins is concurrently 2.5 fold higher. When investigating group II and group I proteins, selected by our strategy it is evident that the percentage of removed proteins is diminished to 9% and 5% respectively, and the percentage of characterized proteins is increased to 65% and 64% respectively. The prevalence of mitochondrial proteins within the number of characterized proteins increases from 6% for the total 2006 dataset to 45%, 58% and 85% for group III, group II and group I respectively.

When increasing the selectivity of the applied prediction models, in our case by focusing on group II and especially group I proteins, it is evident that the probability of finding hypothetical proteins, which will have a function in the predicted sub-cellular compartment is increased. However, it is also evident that the higher the selectivity, the lower the total number of identified proteins with the desired functionality. For group III proteins, 24 out of a total of 67 mitochondrial proteins were discovered. For group I, only 11 proteins out of the 67 mitochondrial proteins were discovered.

The three groups of investigated proteins contain a total of 36 proteins that in November 2008 was still annotated as hypothetical. According to our selection strategy, these proteins are predicted to be expressed and to have roles in a mitochondrial context. To investigate the potential of these proteins, we investigated the protein domains of 6 hypothetical proteins of group I to see if they would suggest a mitochondrial function for the relevant protein (Table 4). 4 out of the 6 proteins each contain one domain that are experimentally characterized in mitochondria and therefore may have putative mitochondrial functions. The four domains were found to be a Complex I-Lyr domain, a Methyltransferase 12 domain, a Sel 1 domain and a DUF1640 domain. The Complex I-LYR domain is present in a family of proteins including the mitochondrial NADH-ubiquinone oxidoreductase complex I. The methyltransferase 12 domain is found in a variety of methyltransferases including one functioning in mitochondria. Sel I like repeats domain is found in a vast amount of proteins including HSP70, HSP90, and in the mitochondrial Tom 70 import receptor. The DUF1640 domain is present in the mitochondrial protein FMP32 found in *Saccharomyces cerevisiae*. [26-30]

**Table 4:** Identified protein domains of 6 hypothetical proteins

**Accession**	**Domain**	**Description of protein domains**
NP_001036096	Complex-1-LYR	This hypothetical protein contains a Complex-1-LYR domain. The domain is present in a family of proteins, which include mitochondrial proteins from NADH-ubiquinone oxidoreductase complex 1. The domain is also present in the *Saccharomyces cerevisiae *protein Isd11, which is located in the mitochondrial matrix associated with the inner membrane. Isd11 protein is a subunit of the mitochondrial Fe/S protein biogenesis [26,27]
NP_077025	Methyltransf 12	Methyltransferase 12 domain is present in proteins, which actively transfer methyl from ubiguitous S-adenosyl-L-methionine (SAM) to nitrogen, oxygen or carbon. This methyltrasferase domain is found in a variety of SAM-dependent methyltransferases including Coq3 methyltransferase, which is a mitochondrial protein involved in ubiquinone biosynthesis. Coq3 protein is located in the matrix of the mitochondria [28,31,32]
NP_055588	Sel1	Sel1 like repeats are tetratricopeptide repeats (TPR) identified in LIN-12 proteins of *Caenorhabditis elegans *as a negative regulator of the Notch pathway [33] TPR-repeats are found in a variety of proteins including eukaryotic chaperone complexes involving HSP70 and HSP90, and TPRs are also present in the mitochondrial Tom70 import receptor [29,30]
EAW75090	DUF1640	DUF1640 domain is found in proteins of unknown functions. In *Saccharomyces cerevisiae *a protein containing the domain is named FMP32 (Found in mitochondrial proteome protein 32) and was localized to the mitochondria. [uniprot.org]
		
NP_612455	DUF143	DUF143: This domain has no known function and is found in the *iojap *protein of maize. The protein has no known function [34]
EAW74251	Trm112p	Trm112p is a zinc finger domain found in the TRM112 protein that is required for tRNA methylation in *Saccharomyces cerevisae*. [35]

Description of protein domains identified in 6 hypothetical proteins of Group I, predicted to be expressed and to have a role in a mitochondrial context. In 4 out of 6 proteins, the identified protein domains have been described in experimentally characterized proteins of the mitochondria (First 4 domains).

To demonstrate that the effectiveness of our *in vitro *selecting strategy is not dependent on neither TargetP nor the SMART program, MITOPRED and WoLF PSORT replaced TargetP and was together with the SMART program, used as basis for our selection strategy in order to screen the 2006 dataset for proteins predicted to be mitochondrial. Similarly, the SMART program was replaced by Prosite and was together with TargetP used as basis for our selection strategy in order to screen a selection of the 2006 dataset (See Additional file 1). Using MITOPRED to screen the 2006 dataset, 807 out of 5860 proteins were predicted to be mitochondrial. Of the 807 proteins, 394 (49%) have been removed, 164 (20%) have been characterized and 35 of these (21%) have been characterized as mitochondrial. Using a combination of MITOPRED and the SMART program, 198 out of 5860 proteins were predicted to be mitochondrial. Of the 198 proteins, 16 (8%) have been removed, 113 (57%) have been characterized and of these 28 (25%) have been characterized as mitochondrial (Table 5).

**Table 5:** Validation of selection strategy using a variety of prediction tools

**Group**	**Resulting proteins**	**Hypothetical proteins**	**Characterized proteins**	**Removed proteins**	**Characterized mitochondrial proteins**
**TargetP + SMART** **(Grp I)**	20	30% (6 of 20)	65% (13 of 20)	5% (1 of 20)	85% (11 of 13)
**TargetP + SMART** **(Grp III)**	100	36% (36 of 100)	53% (53 of 100)	11% (11 of 100)	45% (24 of 53)
**MITOPRED + SMART**	198	34% (68 of 198)	57% (113 of 198)	8% (16 of 198)	25% (28 of 113)
**WoLF PSORT + SMART**	154	31% (48 of 154)	61% (94 of 154)	7% (11 of 154)	28% (26 of 94)
**TargetP + Prosite** **(Reliability class A)**	9	11% (1 of 9)	89% (8 of 9)	0%	100% (8 of 8)

**2006 dataset**	-	25% (1455 of 5860)	21% (1215 of 5860)	54% (3190 of 5860)	6% (67 of 1215)

Different combinations of prediction tools were used on either the whole 2006 dataset or parts of it, to demonstrate that our selection strategy can use a variety of prediction tools and is neither dependent on TargetP nor the SMART program.

Using WoLF PSORT to screen the 2006 dataset, 754 out of 5860 proteins were predicted to be mitochondrial. Of the 754 proteins, 199 (26%) have been removed, 123 (16%) have been characterized and 31 (25%) of these have been characterized as mitochondrial. Using a combination of WoLF PSORT and the SMART program, 154 out of 5860 proteins were predicted to be mitochondrial. Of the 154 proteins, 11 (7%) have been removed, 94 (61%) have been characterized and 26 of these (28%) have been characterized as mitochondrial (Table 5). Using MITOPRED or WoLF PSORT alone to screen the 2006 dataset, respectively 35 and 31 of the total of 67 mitochondrial proteins were identified. However, both MITOPRED and WoLF PSORT did also select 394 proteins that after 2006 have been removed by GenBank. As was the case with TargetP, MITOPRED and WoLF PSORT are efficient in finding mitochondrial proteins, but they are not alone suitable for screening hypothetical proteins for novel translatable candidates. By replacing TargetP with either MITOPRED or WoLF PSORT, our selection strategy is able to identify 26 to 31 of the total number of 67 proteins listed in MitoCarta as characterized mitochondrial proteins. Only 7-8% of the proteins have been removed and the remainder is either characterized or still hypothetical. This demonstrates the efficiency of our *in silico *selection strategy using MITOPRED or WoLF PSORT is comparable to our selection strategy using TargetP.

Of the proteins identified by our selection strategy based on TargetP, up to 85% of the proteins that have been characterized have been characterized as mitochondrial. For our selection strategy based on MITOPRED or WoLF PSORT, the corresponding values are 25% and 28% respectively. The specificity of our selection strategy is therefore dependent on the method of localization prediction used. TargetP relies on the presence of well-defined mitochondrial presequences, whereas MITOPRED and WoLF PSORT rely on putative Pfam domains and signaling motifs.

The SMART program was replaced with the Prosite prediction tool, which together with TargetP was used in our selection strategy to analyze a subset of the 2006 dataset. TargetP predicted 52 of the 5860 proteins to belong to reliability class A. 15 of the 52 proteins have been removed and 14 of the 52 proteins have been characterized as mitochondrial. Only 9 of the 52 proteins were by Prosite found to contain known protein domains. 1 of the 9 proteins is still annotated as being hypothetical, while 8 of the found proteins have been characterized as mitochondrial. Prosite was therefore able to identify 8 of the 14 mitochondrial proteins present in the subsection of the 2006 dataset predicted by TargetP to be in reliability class A and furthermore, none of the removed proteins were selected by Prosite. In comparison, the SMART program was able to identify 11 of the 14 mitochondrial proteins. For the subset of proteins investigated, the efficiency of our selection strategy base on Prosite is therefore comparable with our selection strategy based on the SMART program.

## Conclusion

A hypothetical protein may have a, yet uncharacterized, role in a biological context or simply be the predicted result of a pseudogene and with no biological relevance. In order to screen a dataset of hypothetical proteins, we propose a simple selection strategy where proteins are selected on the basis of well-characterized targeting peptides and protein domains. We have utilized a database of hypothetical proteins dating from 2006 and reviewed their annotated status in 2008. Accordingly, we can verify our selection strategy by reviewing the proteins that were hypothetical in 2006, but have been experimentally characterized by November 2008. We chose to screen for hypothetical proteins predicted to be mitochondrial since considerable amounts of work have been performed within the last couple of years to build extensive databases of the human mitochondrial proteome, summarized in works like MitoCarta. From the 2006 dataset, 5860 hypothetical proteins were identified, and from this dataset, we used TargetP together with the SMART program to identify 100 proteins that we believe, have a high probability of being expressed mitochondrial proteins, based on our selection strategy. This list is not exhaustive as, for instance, hypothetical proteins with mitochondrial internal targeting signals but no mitochondrial presequence, were not considered. When compared to the 2008 dataset, we found that 53 of the 100 hypothetical proteins predicted to be mitochondrial have now been characterized, and 45% of those were found to be mitochondrial. In comparison, only 6% of the characterized proteins from the 2006 dataset have been categorized as mitochondrial in the same time period. Increasing the selectivity of TargetP increases the incidence of characterized mitochondrial proteins to 85%, but unfortunately decreases the total number of mitochondrial proteins identified.

Investigating identified protein domains of 6 of the 36 hypothetical proteins predicted to be mitochondrial, we found a subset of 4 proteins having a strong mitochondrial signature in their identified protein domains. It is our opinion that these proteins are very interesting candidates for further experimental characterizations.

In present work we have applied our selection strategy in the search for human mitochondrial proteins using TargetP and the SMART program. From the characterized proteins we were able to verify the fidelity of our *in silico *selection strategy.

By using different combinations of prediction tools, we demonstrated that our selection strategy is general. The possibility of selecting different prediction tools thereby allows the identification of hypothetical proteins with a high probability of having a role in any organelle compartment where the internal targeting signals are characterized. Furthermore, many of the targeting signals and identifiable protein domains that are valid in human cells, are also valid in other eukaryotes. Our selection strategy can therefore be applied on a wide array of organisms.

## Authors' contributions

CD and PS contributed the initial concept for this work. CD, PS, MS, MR, and LJR participated in the design of the study and performed the analyses. Paper was written with insights from the other authors. All authors read and approved the final manuscript.

## Supplementary Material

Additional file 1 **2006 & 2008 Dataset**. Protein entries of the 2006 and 2008 dataset. Results from our *in silico *selection strategy. Click here for file
